# Tilianin attenuates inflammasome activation in endothelial progenitor cells to mitigate myocardial ischemia-reperfusion injury

**DOI:** 10.1371/journal.pone.0311624

**Published:** 2024-10-10

**Authors:** Miaomiao Wang, Jiapeng Li, Xu Hu, Mengmeng Fu, Xiaoxue Li, Davaadagva Damdinjave, Ming Xu, Ruifang Zheng, Jianguo Xing

**Affiliations:** 1 Key Laboratory of Xinjiang Phytomedicine Resources for Ministry of Education, School of Pharmacy, Shihezi University, Shihezi, China; 2 Xinjiang Institute of Materia Medica, Xinjiang Key Laboratory of Uygur Medicine, Urumqi, China; 3 China Pharmaceutical University, Nanjing, China; 4 Department of Cardiology, Zhongda Hospital, School of Medicine, Southeast University, Nanjing, China; 5 School of Pharmacy, Mongolian National University of Medical Sciences, Ulaanbaatar, Mongolia; ABEx Bio-Research Center, BANGLADESH

## Abstract

Tilianin (TIL), a bioactive component derived from *Dracocephalum Moldavica* L., has been recognized for its anti-inflammatory properties. However, its effects on the Nlrp3 inflammasome within endothelial progenitor cells (EPCs) during myocardial ischemia-reperfusion injury (MIRI) remain unexplored. This study aimed to elucidate the role of TIL in modulating Nlrp3 inflammasome activation under MIRI conditions. A mouse model of MIRI was established to assess the therapeutic potential of TIL. EPCs treated with TIL at concentrations of 5, 10, and 20 μM were administered into the myocardium before reperfusion. Additionally, the cardioprotective effects of TIL were further examined by pre-treating EPCs with the compound before exposing them to hypoxia/reoxygenation (H/R) using cardiomyocyte supernatants. The impact on Nlrp3 inflammasome was assessed through western blotting, immunofluorescence, and ELISA. Our results showed that TIL concentration-dependently inhibited Nlrp3 inflammasome-related protein levels,and inhibited Asc oligomerization and Asc-Speck complex formation in EPCs, resulting in improved the migratory capacity and vascular structure formation of EPCs. In addition, TIL-treated EPCs significantly attenuated I/R injury and improved cardiac function. These results suggest that TIL ameliorates the inflammatory response in EPCs by suppressing Nlrp3 inflammasome activation, thereby facilitating neovascularization in the myocardium and conferring protection against MIRI. The study provides valuable insights into the potential of TIL as a therapeutic agent for cardiovascular diseases linked to ischemia-reperfusion injury.

## 1 Introduction

Cardiovascular disease (CVD) is a major disease that seriously jeopardizes human health and has become the number one killer with the highest cause of human death [1]. According to statistics, about one-third of the world’s annual deaths are due to myocardial ischemic disease, and its lethality is the highest in the world [2]. Myocardial ischemia-reperfusion injury (MIRI), which is defined as myocardial injury caused by the restoration of blood flow to previously ischemic myocardium, is an important risk factor in the pathological process of cardiovascular disease [3]. Although reperfusion is the most effective method to improve myocardial ischemia survival, reperfusion may exacerbate myocardial injury, triggering a variety of pathological responses and leading to cardiomyocyte death [4].

Vascular endothelial progenitor cells (EPCs) are a class of circulating cells with important roles in vascular repair and cardiovascular disease treatment, with less peripheral origin, mainly from bone marrow, which are precursor cells of endothelial cells. It has also been reported that EPCs have the ability to differentiate toward mature vascular endothelial cells, which are pluripotent stem cells capable of adhering to, proliferating, and differentiating into vascular endothelial cells to repair damaged blood vessels, as well as forming new vascular structures in the form of vascular neovascularization [5–7], which demonstrates a great therapeutic potential in decreasing myocardial infarction area, reversing ventricular remodeling, and improving cardiac function [8–10]. Therefore, the role of EPCs in maintaining endothelial structural integrity and endothelial function, as well as in neovascularization, is increasingly being emphasized. Studies have confirmed that the number and activity of circulating EPCs affect vascular endothelial function [11]. However, as coronary artery disease becomes more and more severe, circulating EPCs will gradually decrease [12,13]. EPCs have a wandering character and are released from the bone marrow into the bloodstream when the body is injured, and then proliferate, migrate, and homing to the site of injury to repair the vascular endothelium [14]. Previous studies have shown that ex vivo transplanted EPCs restore endothelial function at the site of injury and promote neovascularization [15,16], and exogenous transplantation of EPCs has gradually become a new cell therapy for the treatment of cardiovascular disease [17–19]. Therefore, we hypothesized that ex vivo transplantation of EPCs to promote myocardial neovascularization may be an effective way to ameliorate MIRI.

The NLRP3 Inflammasome has been found to play an important role in the inflammatory response to MIRI [20]. It consists of a nucleotide-binding oligomerization structural domain-like receptor with a Pyrin structural domain (NLRP3), apoptosis-associated speck-like protein (ASC), and cysteine-aspartate-specific protease 1 (caspase-1), where ASC proteins serve as a bridge connecting receptor proteins and effector proteins, and the assembly of a variety of inflammasome requires ASC oligomerization [21]. NLRP3 inflammasome are intracellular multiprotein complexes that are activated in response to a variety of stimuli, including bacteria, viruses, extracellular ATP, pollutants, metabolic dysregulation, and tissue injury [22–24]. After activation, ASC and pro-caspase-1 are subsequently attracted to assemble into inflammasome with diameters of up to micrometers. This induces caspase-1 self-cleavage and activation, and the activated caspase-1 cleaves IL-1β into active IL-1β for release to trigger an inflammatory response. It has been shown that the sustained activation of NLRP3 inflammasome is involved in the course of a variety of inflammatory diseases that pose serious risks to human health, such as MIRI, alzheimer’s disease, atherosclerosis, gout, and so on [25–30]. Therefore, NLRP3 inflammasome may be a therapeutic target for I/R injury. Our team’s work over the years has identified the activation of NLRP3 Inflammasome as a mechanism for initiating vascular endothelial inflammatory responses, which can lead to coronary artery inflammation in early obesity and vascular endothelial impairment in diabetes [31–33]. It has been shown that NLRP3 Inflammasome activation are not only involved in cellular injury caused by various factors, but also have a role in regulating endothelial precursor cell function [34]. Studies have reported that Salvianolic Acid B and Astragaloside IV can protect the function of EPCs via NLRP3 Inflammasome [35,36]. It has also been shown that EPCs protect against renal ischemia-reperfusion injury by inhibiting NLRP3 Inflammasome activation [37]. Therefore, we believe that EPCs and NLRP3 Inflammasome are interrelated, but the relationship between NLRP3 Inflammasome and EPCs in MIRI has not been studied.

Tilianin (TIL) is an active monomer isolated from *Dracocephalum Moldevica* L. and belongs to the flavonoid group. TIL has various pharmacological properties such as cardioprotection, anti-inflammatory, neuroprotective, antihypertension and antioxidant effects [38]. Studies have shown that TIL has a significant improvement effect on hypertension, hyperlipidemia, atherosclerosis and other pathological states in model rats, and has a significant protective effect on MIRI in model rats [39–42], which has a broad application prospect in the prevention and treatment of cardiovascular and cerebrovascular diseases. Recent studies have shown that TIL induces pharmacological effects through anti-inflammatory actions. NLRP3 inflammasome as a molecular switch to regulate inflammation generation has become a top priority in the field of natural immunity research in recent years. In diabetic cardiomyopathy (DCM), the combination of TIL and Syringin significantly reduced the expression of NLRP3, TNF-α, IL-1β, and IL-6 and attenuated myocardial injury [43]. However, whether the effect of TIL on NLRP3 inflammasome activation in EPCs is unknown.

In the current study, by detecting cellular functions, Nlrp3 inflammasome-related indexes, cardiac functions, and histological studies, we investigated the improvement of angiogenesis in the context of MIRI by TIL through the modulation of Nlrp3 inflammasome activation in EPCs from the point of view of the regulation of EPCs function by Nlrp3 inflammasome. Our findings provide new attempts to treat stem cell transplantation and new insights into TIL against MIRI.

## 2 Materials and methods

### 2.1 TIL and animals

TIL (purity > 98%, lot number 20220105) was sourced from the Xinjiang Institute of Materia Medica (Urumqi, China). Male C57BL/6 mice, aged 6–8 weeks and weighing 20–22 g, were obtained from GemPharmatech (Nanjing, China). The animals were maintained under a 12 h light/dark cycle at a controlled temperature of 25°C ± 5°C and a humidity of 55–60%. They were provided with standard laboratory food and water ad libitum. All experimental procedures involving animals were reviewed and approved by the Ethics Committee of the Chinese University of Pharmacy and Pharmaceutical Sciences. These procedures were in strict accordance with the Guidelines for the Care and Use of Laboratory Animals as stipulated by the European Parliament (Directive 2010/63/EU).

### 2.2 Cell extraction, culture and characterization

The experimental details are provided in the online Supplemental Information, include Immunofluorescence identification of primary cardiomyocytes (S1 Fig) and endothelial progenitor cells (S2 Fig) and Knockdown of Nlrp3 in EPCs of WT mice (S3 Fig and S1 Appendix).

### 2.3 CCK-8

EPCs in logarithmic growth phase were taken, and the cell density was adjusted to 1×105 cells/mL with FBS-containing medium, and 100 μL per well was inoculated into 96-well culture plates. After cell apposition the culture medium was changed to TIL concentrations of 0, 1, 2, 5, 10, 20, 40 and 80 μM for 24 h of incubation. The absorbance (OD value) of each well was determined and cell viability was calculated according to the Cell Counting Kit-8 (CCK-8, Target Mol, Shanghai, China) assay.

### 2.4 Functional testing of EPCs

#### 2.4.1 Scratch wound assay

Cells were cultured in six-well plates, grown until confluence, and incubated in serum-deficient medium containing different concentrations of TIL (5, 10, and 20 μM) or OLT1177 (10 μM) for 24 h. After washing with PBS and gently scratching with a sterile pipette tip, H/R primary cardiomyocyte supernatants were added to the medium and incubated for 24 h. Photographs of the injured area were taken immediately after the scratch 0 h and 24 h after scratch. The trauma area was calculated using Image J (NIH, Littleton, CO, USA), and the closure rate was quantified as the percentage of the recovered area to the initial trauma area.

#### 2.4.2 Transwell migration assay

The transwell migration assay was performed with an 8 μm pore size NEST culture plate insert (NEST, Wuxi, China). Specifically, EPCs in logarithmic growth phase were taken and incubated in serum-deficient medium containing different concentrations of TIL (5, 10, and 20 μM) or OLT1177 (10 μM) for 24 h. EPCs (3×105 cells/mL) were then suspended in 200 μL of H/R primary cardiomyocyte supernatants (without FBS) and added to the upper chamber, and the lower chamber was filled with normal medium (with FBS). After 24 h of incubation, non-migrated cells were gently removed with a cotton swab. The migrated cells were fixed in 4% paraformaldehyde solution for 15 min and stained with 0.1% crystal violet for 5 min, and the stained cells were photographed and counted under an inverted microscope (Shanghai Tow Intelligent Technology Co., China).

#### 2.4.3 Tube formation assay

A tube formation assay was used to evaluate the formation of capillary-like structures in vitro using Matrigel (Mogengel Biotech, Nanjing, China). Briefly, 96-well culture plates were coated with Matrigel (50 μL/well) at 37°C for 1 h. EPCs (3×10^4^ cells/well) with different treatments were seeded onto the Matrigel and cultured at 37°C. Tube formation was quantified after 4 h using an inverted microscope (Jiangnan, XD-202, China).

### 2.5 ASC oligomerization assay

EPCs were washed with ice-cold PBS, and 500 μL of ice-cold buffer (NP-40: PMSF = 100:1) was added, and gently shaken to spread the bottom of the culture flask. Cells were lysed at 4°C for 30 min, lysates were centrifuged at 6000 ×g for 15 min at 4°C, and the supernatant was added to 5×SDS loading buffer at a ratio of 4:1 to make whole cell lysate. The microspheres were resuspended in 500 μL of ice-cold PBS, washed by slight vortexing, centrifuged at 6000 ×g for 15 min at 4°C, the supernatant was removed, and crosslinked by adding 25 mM disuccinimidyl suberate (DSS, Sangon Biotech, Shanghai, China), and heated in a metal bath at 37°C for 30 min, with vortexing every 10 min to crosslink the proteins sufficiently, and the last vortex was not necessary. The samples were then centrifuged at 6000 ×g for 15 min at 4°C. The supernatant was removed, 100 μL of 1× SDS loading buffer was added, and the samples were boiled in a metal bath at 100°C for 15 min for western blotting detection. The supernatant was removed, and the crosslinked pellets were resuspended in 100 μL of 1× SDS loading buffer. The samples were boiled for 15 min at 100°C and analyzed by western blotting.

### 2.6 ELISA

Cell supernatants were collected and lactate dehydrogenase (Ldh) levels were determined by microplate assay using a commercial kit (Jiancheng Bioengineering, Nanjing, China). In addition, we determined the level of Il-1β by a commercial enzyme-linked immunosorbent assay (ELISA) kit (ABclonal, Wuhan, China).

### 2.7 RNA Isolation and Quantitative Reverse Transcription Polymerase Chain Reaction (qRT-PCR)

Total RNA was extracted using TRIzol reagent (Vazyme Biotech Co., Ltd) according to the manufacturer’s protocol. RNA concentration was determined using a NanoDrop 2000 spectrophotometer (Thermofisher, NanoDrop^TM^ OneC, USA), and samples with an A260/A280 ratio of 1.8 ~ 2.0 were taken for reverse transcription. Reverse transcription was performed using the Evo M-MLV Reverse Transcription Premixed Kit (with gDNA Removal Reagent) Ver.2 (Accurate Biology, Hunan, China). Detection was performed using the SYBR Green Pro Taq HS premixed qPCR kit (containing ROX), (Accurate Biology, Hunan, China). PCR thermocycling conditions were 95°C for 30 s, 95°C for 10 s, and 60°C for 30 s for a total of 40 cycles. The relative expression of mRNA was normalized to β-actin and calculated using the standard 2- (ΔCt sample-ΔCt control) method. Primers used for PCR are shown in Table 1.

**Table 1:** Primers used for PCR.

Gene	Forward	Reserve
*Aim2*	GTCACCAGTTCCTCAGTTGTG	CACCTCCATTGTCCCTGTTTTAT
*Nlrc4*	GAAACACTGTACGATCAGCTCC	CATGTTCTTGAAGCGATGGTTTT
*Nlrp3*	ATTACCCGCCCGAGAAAGG	TCGCAGCAAAGATCCACACAG
*Tnf-α*	CCCTCACACTCAGATCATCTTCT	GCTACGACGTGGGCTACAG
*Il-6*	TAGTCCTTCCTACCCCAATTTCC	TTGGTCCTTAGCCACTCCTTC
*Il-1β*	GCAACTGTTCCTGAACTCAACT	ATCTTTTGGGGTCCGTCAACT
*Il-1α*	CGAAGACTACAGTTCTGCCATT	GACGTTTCAGAGGTTCTCAGAG
*β-actin*	CTACCTCATGAAGATCCTGACC	CACAGCTTCTCTTTGATGTCAC

### 2.8 Western blotting analysis

The protein expression of the cells and the cell supernatants was analyzed using western blotting as described previously [44]. Unlike cellular protein extraction, protein extraction from cell supernatants is first performed by adding equal amounts of methanol and one-quarter volume of chloroform to the cell culture supernatant. After spin centrifugation, the upper solution is removed and equal amounts of methanol are added. After spin centrifugation, the protein precipitate was added to Triton loading (Triton buffer: 1×SDS protein electrophoresis buffer). Protein concentrations were quantified by using a BCA protein quantity kit (Beyotime, Shanghai, China). Equal amounts of cell lysate or tissue protein were separated by sodium dodecyl sulfate-polyacrylamide gel electrophoresis (SDS-PAGE), and then transferred to 0.45 μm polyvinylidene difluoride membrane (PVDF, Millipore, USA). After being blocked with 5% non-fat milk diluted in Tris-buffered saline with Tween-20 (TBST, 100 mM Tris-HCL, pH 7.4) for 2 h, the membranes were incubated with each primary antibody (1:100–1:5000 dilutions) overnight at 4°C. After washing three times with TBST, the membranes were incubated with goat anti-rabbit IgG (1:5000, Abways Technology, Inc., Shanghai, China) for 1.5 h. The blottings were detected using an automated chemiluminescence image analysis system (5200 Multi, Tanon, China, Shanghai, China) with LumiGlo and hydrogen peroxide (1:1, Tanon, Shanghai, China) using β-actin protein as a reference, and staining intensity was analyzed using Image J software (NIH, Littleton, CO, USA).

### 2.9 Myocardial Ischemia/Reperfusion (I/R) surgery and treatment in mice

Male C57 BL/6 mice (6 to 8 weeks of age and weighing 20 to 23g) were used to establish a mouse MIRI model by coronary artery ligation [45]. Briefly, mice were anesthetized with 1.25% (MACKLIN, Shanghai, China) tribromoethanol, respiratory support was established with a small animal ventilator (Tawang Intelligent Technology Co, Shanghai, China), and body temperature was maintained at 37°C with a heating pad. The chest was opened in a lateral left axillary position under the microscope, bluntly opened between 2–3 helpers, and the pericardium was gently torn with micro forceps to expose the pulmonary artery cones and the left auricle. Ligation was performed 2 mm below the midpoint of the arterial cone and the left auricle, with a needle depth of 1 mm and a width of l-2 mm, and the left anterior descending branch was ligated with a 6–0 nondestructive suture, with moderate ligation strength and a live knot, and the myocardium at the location of the ligature and below it became whitened and the movement was weakened, suggesting that the modeling had been successful. Ischemia for 30 min. EPCs were injected intramyocardially before reperfusion. During surgery, a heating pad was used to help maintain the body temperature at 37°C. The mice in the sham-operated group were only threaded with 6–0 surgical wires at the corresponding locations without ligation, and the rest of the procedure was the same as that in the model group and the treatment group. After surgery, the mice were kept warm and given respiratory support until spontaneous respiration was restored and they were able to eat normally.

### 2.10 Echocardiography detection

Mice were anesthetized with 1.25% tribromoethanol on days 7 and 28 after MIRI, and a Vevo 3100 LT high-frequency color ultrasound system (FUJIFILM, VisualSonics, Japan) and M-mode echocardiography were used to measure the left ventricular ejection fraction (LVEF), left ventricular short-axis shortening (LVFS), left ventricular diastolic end volume (LVEDV) and left ventricular end-systolic volume (LVESV).

### 2.11 Myocardial injury biomarkers assay

Whole blood was taken from mice on postoperative day 28. Serum was isolated from the blood samples. Aspartate Transaminase (Ast) levels were determined using the glutamic oxal transaminase (Ast/Got) test kit (Jiancheng Bioengineering, Nanjing, China), and mouse cardiac-specific troponin T (cTnT) levels were determined using a commercial enzyme-linked immunosorbent assay (ELISA) kit (Meimian, Jiangsu, China) according to the instructions.

### 2.12 Histological studies

#### 2.12.1 Assessment of infarct size

Mouse hearts were taken 28 days after MIRI and cut into five 1-mm-thick transverse slices from the apex to the base perpendicular to the LAD branch of the coronary artery, and the slices were immediately stained by immersion in 1% red tetrazolium (TTC, Beijing, China) in the dark at 37°C for 15 min to distinguish infarcted tissue from normal myocardium. Photographs were taken and the area of white infarcted area was calculated using Image-Pro Plus (Ipp) software (version 6.0.0.260, Media Cybernetics Corporation, USA).

#### 2.12.2 Histopathological analysis

Paraffin-embedded fixed mouse hearts were prepared in 8 μm thick sections. According to the manufacturer’s instructions, tissue sections were stained for histopathological damage using Hematoxylin and Eosin (HE) Staining kit (SenBeiJia Biological Technology Co., Nanjing, China) and Masson’s Trichrome Staining kit (Solarbio, Beijing, China). Cardiac histologic damage was assessed by quantitatively measuring tissue damage under blinded observation. Photographs were taken and the area of blue infarcted area was calculated using Image-Pro Plus (Ipp) software (version 6.0.0.260, Media Cybernetics Corporation, USA).

#### 2.12.3 Immunofluorescence staining

Mouse hearts 28 days after MIRI were taken, and cryosections (8 μm thickness) of hearts embedded with optimal cutting temperature compound (OCT, SAKURA, Japan) were prepared for immunofluorescence staining. Specifically, frozen sections were fixed with cold acetone for 15 min, then permeabilized with 0.3% (v/v) Triton X-100 (Solarbio, Beijing, China) in PBS for 15 min, blocked with 1% (w/v) bovine serum albumin (BSA, Yuanye Bio-Technology Co., Shanghai, China) for 1 h, and then immunofluorescently stained with Cd31 (1:150, Abways Technology, Inc., Shanghai, China) overnight at 4°C, and then incubated with Cy3-coupled goat anti-mouse IgG (H+L) (1:200, Proteintech™, Wuhan, China), respectively, for 2 h. The nuclei were stained with Dapi.

The treated EPCs were fixed and blocked as described above, then incubated with Asc (1:100, Bioword Technology, Inc, Shanghai, China), followed by Cy3-coupled goat anti-rabbit IgG (H+L) incubation and Dapi counterstaining. Finally, fluorescence images were taken by a laser scanning confocal microscope (ZEISS Scope. A1, Germany). Image processing was performed using ZEN blue 2.3 software (Carl Zeiss, Germany).

### 2.13 Statistical analysis

Data are expressed as mean ± standard deviation (SD) and were obtained in three or more independent experiments. GraphPad Prism 9.4.0 was used for statistical analysis. Comparisons between multiple groups were performed using one-way analysis of variance (ANOVA), and differences were considered statistically significant at P < 0.05.

## 3 Results

### 3.1 Cytotoxicity validation of TIL

**Fig 1:**
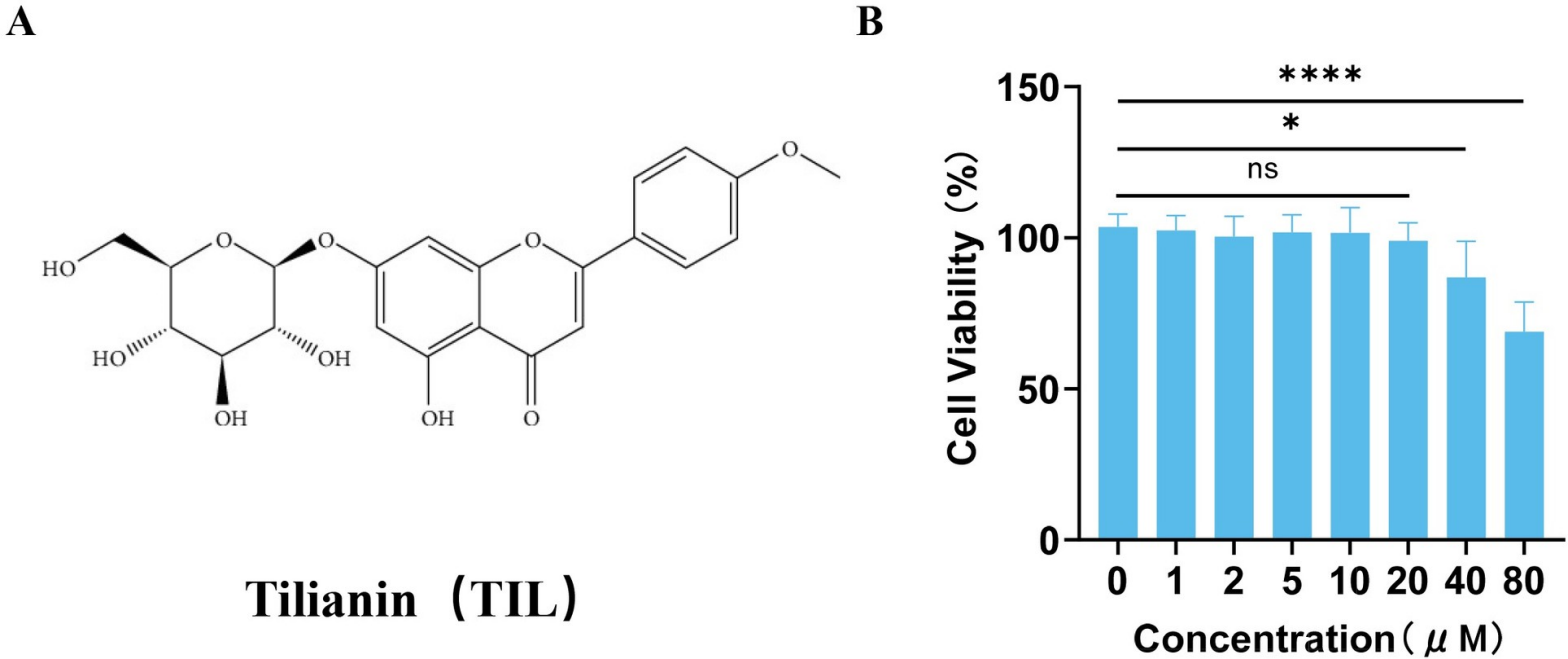
Toxicity of different concentrations of TIL on EPCs. (A) Structure of TIL. (B) Effect of different concentrations of TIL on EPCs viability. Results are represented as mean ± SD. * denotes P < 0.05, ** P < 0.01, *** P < 0.001, **** P < 0.0001.

To assess the potential toxicity of TIL on EPCs, the cells were incubated with various concentrations of TIL for 24 h. The viability of the EPCs was evaluated using a CCK-8 assay. The results indicated that TIL concentrations up to 20 μM did not significantly impact EPCs viability, suggesting minimal toxicity towards EPCs. However, at concentrations above 20 μM, there was a notable decrease in cell viability (Fig 1A and 1B). Based on these results, TIL concentrations of 5 μM, 10 μM, and 20 μM were selected for further experiments.

### 3.2 H/R primary cardiomyocyte supernatants induce activation of Nlrp3 Inflammasome in EPCs

**Fig 2:**
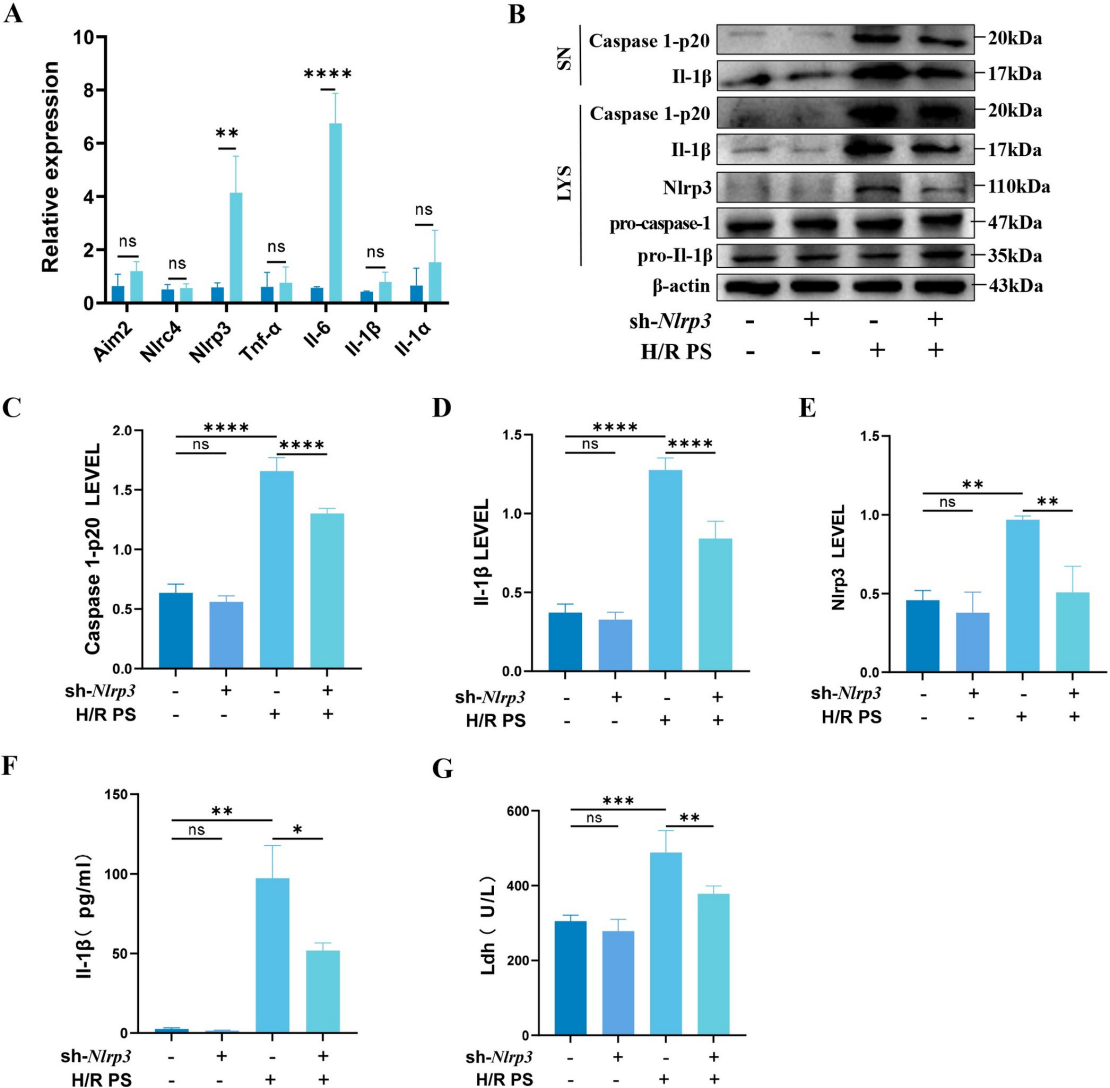
Effect of H/R PS on Nlrp3 Inflammasome. H/R PS represents supernatant from H/R primary cardiomyocytes. (A) qRT-PCR to detect the expression of inflammatory factors. (B) Western blotting was performed to analyze the levels of cleaved Il-1β and caspase 1-p20 in cultured SN as well as Nlrp3, caspase 1-p20, Il-1β, pro-Il-1β, pro-caspase-1, and β-actin in the lysates (Input) of EPCs. (C-E) Quantitative analysis of Nlrp3, caspase 1-p20, and Il-1β gray values. (F-G) Levels of Il-1β and Ldh in cell supernatants. Results are expressed as mean ± SD, * denotes *P* < 0.05, ** *P* < 0.01, *** *P* < 0.001, **** *P* < 0.0001.

The expression of inflammatory genes was evaluated using qRT-PCR following the incubation of EPCs with H/R primary cardiomyocyte supernatants. A significant upregulation of Nlrp3 was observed (Fig 2A), suggesting a potential link between the cellular inflammatory response and Nlrp3 inflammasome activation induced by H/R primary cardiomyocyte supernatants. Subsequent analysis via western blotting revealed a notable increase in the expression levels of Nlrp3, Il-1β, and caspase 1-p20 (Fig 2B–2E and S1 Appendix). Conversely, silencing of *Nlrp3* led to a significant reduction in these protein expressions. Further, the concentrations of Il-1β and Ldh in the cell supernatants were quantified using ELISA kits, showing substantial decreases post-*Nlrp3* silencing (Fig 2F and 2G). These results indicated that H/R primary cardiomyocyte supernatants could activate the Nlrp3 inflammasome.

### 3.3 Nlrp3 inflammasome activation affected angiogenesis in EPCs

**Fig 3:**
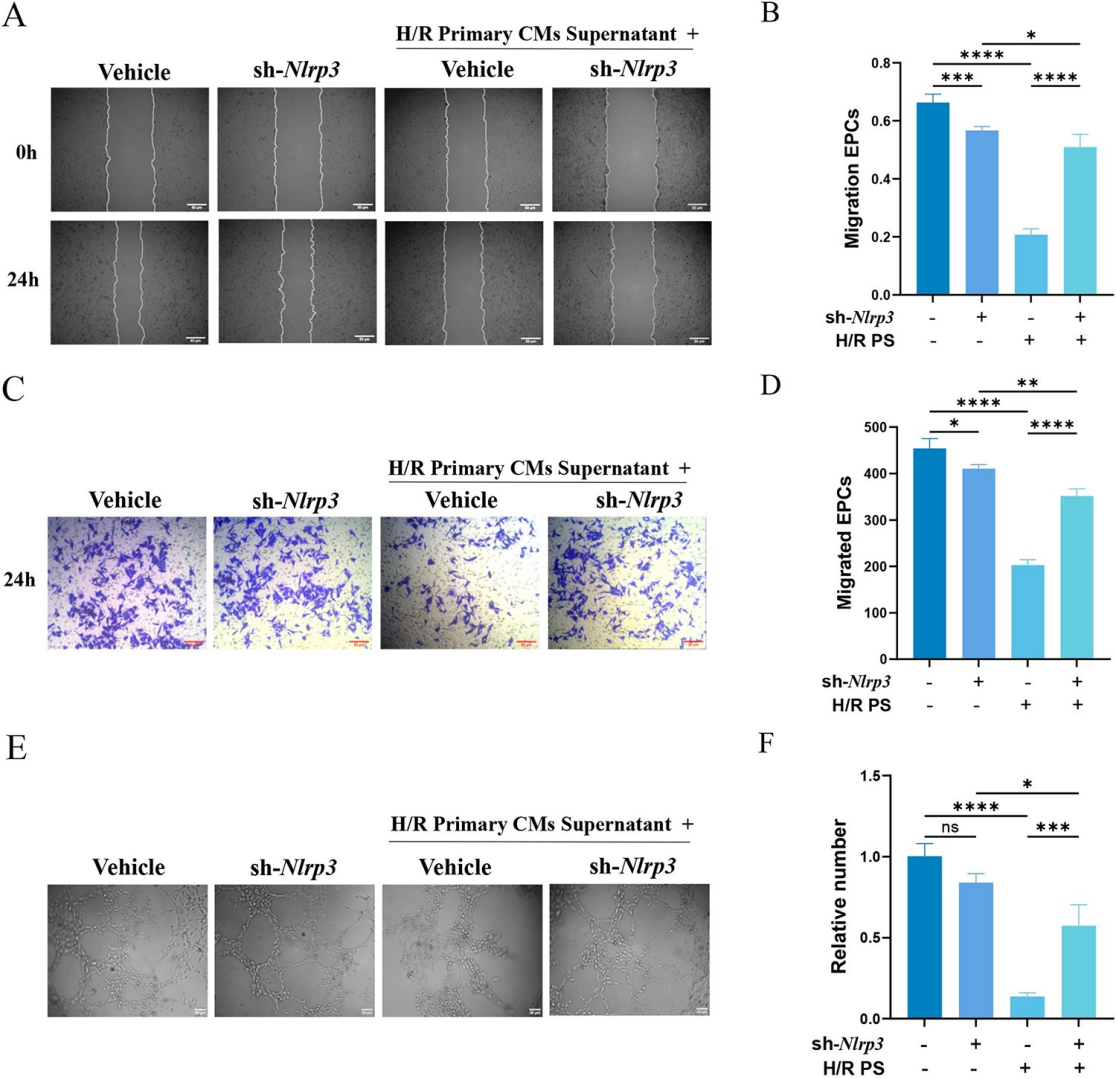
Nlrp3 Inflammasome are activated by H/R PS stimulation and affect EPCs function. H/R PS represents supernatant from H/R primary cardiomyocytes. (A, B) Effect of silencing *Nlrp3* on the migratory capacity of EPCs in scratch assays. (C, D) Effect of silencing *Nlrp3* on the migratory capacity of EPCs in transwell migration assays. (E, F) Effect of silencing *Nlrp3* on the tube-forming ability of EPCs in vitro in vascular-like structure formation assays. Results are represented as mean ± SD. * denotes *P* < 0.05, ** *P* < 0.01, *** *P* < 0.001, **** *P* < 0.0001.

To explore the impact of MIRI on the neovascularization capacity of EPCs, we simulated the MIRI environment in vitro and conducted a series of assays, including scratch wound and transwell migration assays. Our findings revealed that exposure to H/R primary cardiomyocyte supernatants significantly reduced the migratory capacity of EPCs. However, silencing *Nlrp3* significantly enhanced their migratory ability (Fig 3A–3D), likely due to the inhibition of Nlrp3 inflammasome activation. In vascular structure formation experiments, we observed that stimulation with H/R primary cardiomyocyte supernatants caused EPCs to aggregate into sheets or lay flat on the matrix gel, preventing them from forming normal tubular structures. Conversely, when *Nlrp3* was silenced, EPCs were able to form intact tubular networks (Fig 3E and 3F). These results are consistent with our previous observations, further supporting the role of Nlrp3 inflammasome in impairing the neovascularization capacity of EPCs under MIRI conditions.

### 3.4 Nlrp3 inflammasome activation in EPCs affects cardiac function after MIRI in mice

**Fig 4:**
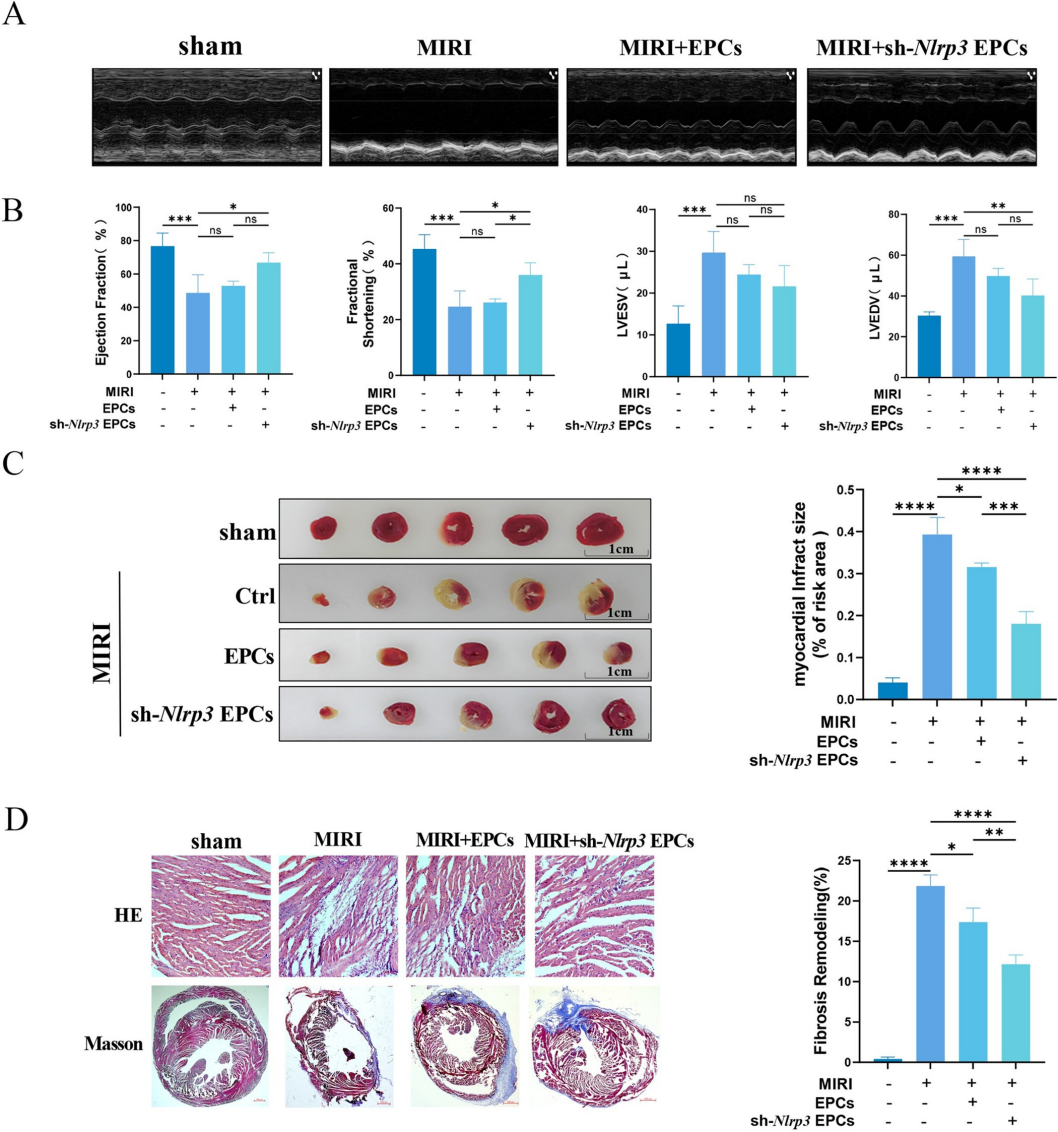
Effects of sh-*Nlrp3* EPCs on cardiac function in MIRI mice. (A) Echocardiograms of each group. (B) Including LVEF, LVFS, LVEDV, and LVESV. (C) Representative images of the heart after TTC staining, with non-infarcted tissue xstained in red and infarcted tissue stained in white. (D) HE staining results showed that sh-*Nlrp3* EPCs improved histopathologic damage after MIRI. (E) Masson’s trichrome staining results showed that myocardial collagen fibers stained blue and myocardial fibers stained red. Results are represented as mean ± SD. * denotes *P* < 0.05, ** *P* < 0.01, *** *P* < 0.001, **** *P* < 0.0001.

Our in vitro findings suggested that the impact of MIRI on the neovascularization capacity of EPCs might be related to the activation of the Nlrp3 inflammasome. To further validate this hypothesis, we investigated the effects of sh-*Nlrp3* EPCs on MIRI in mice. Echocardiographic analysis revealed that in the MIRI group, LVEF and LVFS were significantly reduced, while LVEDV and LVESV were markedly increased. In contrast, the sh-*Nlrp3* EPCs group showed improved cardiac function, with increased LVEF and LVFS and decreased LVEDV and LVESV (Fig 4A and 4B). TTC staining indicated a significant increase in myocardial infarct area in the MIRI group, whereas the infarct area was notably reduced in the sh-*Nlrp3* EPCs group (Fig 4C). Histological examination through HE staining showed disorganized myocardial cells and extensive inflammatory cell infiltration in the MIRI group. These pathological changes were significantly mitigated in the sh-*Nlrp3* EPCs group (Fig 4D). Furthermore, Masson staining demonstrated a reduction in collagen fiber content and a decrease in fibrosis in the sh-*Nlrp3* EPCs group (Fig 4D). These findings are consistent with our initial speculations, further supporting the role of Nlrp3 inflammasome activation in the adverse effects of MIRI on EPC-mediated neovascularization.

### 3.5 TIL concentration-dependent inhibition of Nlrp3 inflammasome activation in EPCs

**Fig 5:**
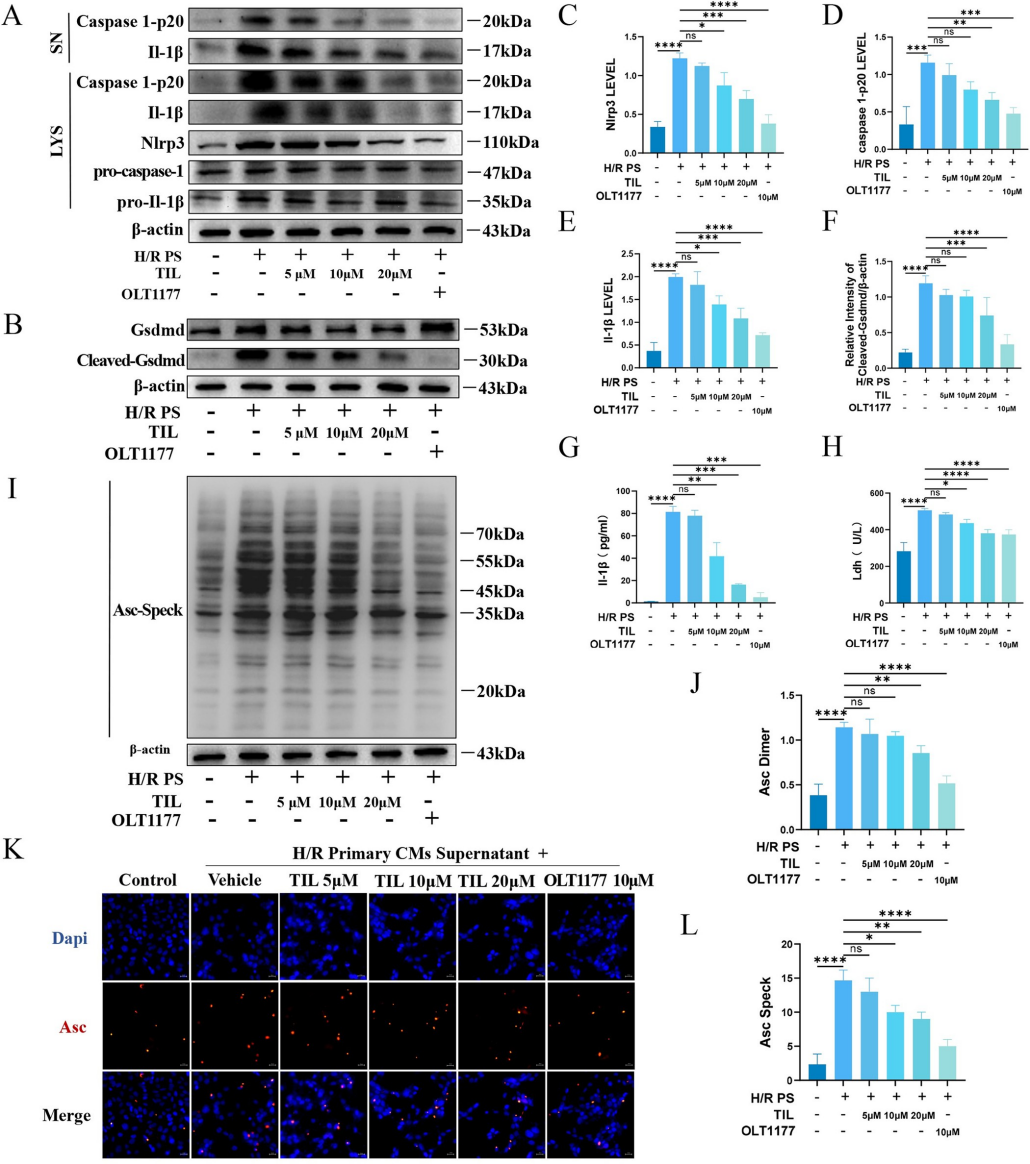
TIL inhibits Nlrp3 inflammasome activation in EPCs. H/R PS represents supernatant from H/R primary cardiomyocytes. (A) EPCs were treated with different concentrations of TIL (5, 10, and 20 μM) or OLT1177 (10 μM) for 24 h before induction of EPCs with H/R primary cardiomyocyte supernatant for 24 h. Western blotting analysis of cleaved Il-1β and caspase 1-p20 levels in culture SN and Nlrp3, pro-Il-1β, pro-caspase-1, and β-actin in lysates (Input) of EPCs. (B) EPCs were treated with different concentrations of TIL (5, 10, and 20 μM) or OLT1177 (10 μM) for 24 h before induction of EPCs with H/R primary cardiomyocyte supernatant for 24 h. Western blotting analysis of Gsdmd, or Cleaved-Gsdmd in lysates. (C-F) caspase 1-p20, Il-1β, Nlrp3, Cleaved-Gsdmd gray values were quantified. (G-H) Il-1β (G) or Ldh (H) production in SN was assessed by ELISA. (I-J) Western blot and quantification of cross-linked Asc in NP-40 insoluble microspheres were performed with anti-Asc antibody. (K-L) EPCs were treated with different concentrations of TIL (5, 10, and 20 μM) or OLT1177 (10 μM), respectively, for 24 h before induction of EPCs with H/R primary cardiomyocyte supernatants for 24 h. Immunofluorescence was used to analyze the formation of Asc spots in EPCs. Results are represented as mean ± SD. * denotes *P* < 0.05, ** *P* < 0.01, *** *P* < 0.001, **** *P* < 0.0001.

To identify anti-inflammatory compounds targeting the Nlrp3 inflammasome, we investigated whether TILcould inhibit p20 cleavage and IL-1β secretion in EPCs to verify its inhibitory effect on inflammasome activation. Our results demonstrated that TIL inhibited Nlrp3 expression, p20 cleavage, and IL-1β release in a dose-dependent manner (Fig 5A, 5C–5E and S1 Appendix). Additionally, TIL concentration-dependently suppressed the inflammasome-induced activation of Gsdmd in EPCs (Fig 5B and 5F and S1 Appendix). Similar inhibitory effects of TIL were observed on Ldh release and Il-1β expression (Fig 5G and 5H).

We further explored the impact of TIL on Asc oligomerization, a crucial step in inflammasome activation. Our findings revealed that TIL inhibited Asc oligomerization in a concentration-dependent manner (Fig 5I and 5J and S1 Appendix). Moreover, during inflammasome activation in EPCs, Asc oligomerization leads to the formation of a large speck in the perinuclear area. Pretreatment with TIL significantly reduced the formation of these Asc specks (Fig 5K and 5L). Since Asc oligomerization is critical for the assembly of Nlrp3 inflammasomes and the subsequent activation of caspase-1, these findings collectively suggest that TIL inhibits Nlrp3 inflammasome activation by preventing the assembly of its components.

### 3.6 TIL improves migratory and angiogenesis capacity of EPCs

**Fig 6:**
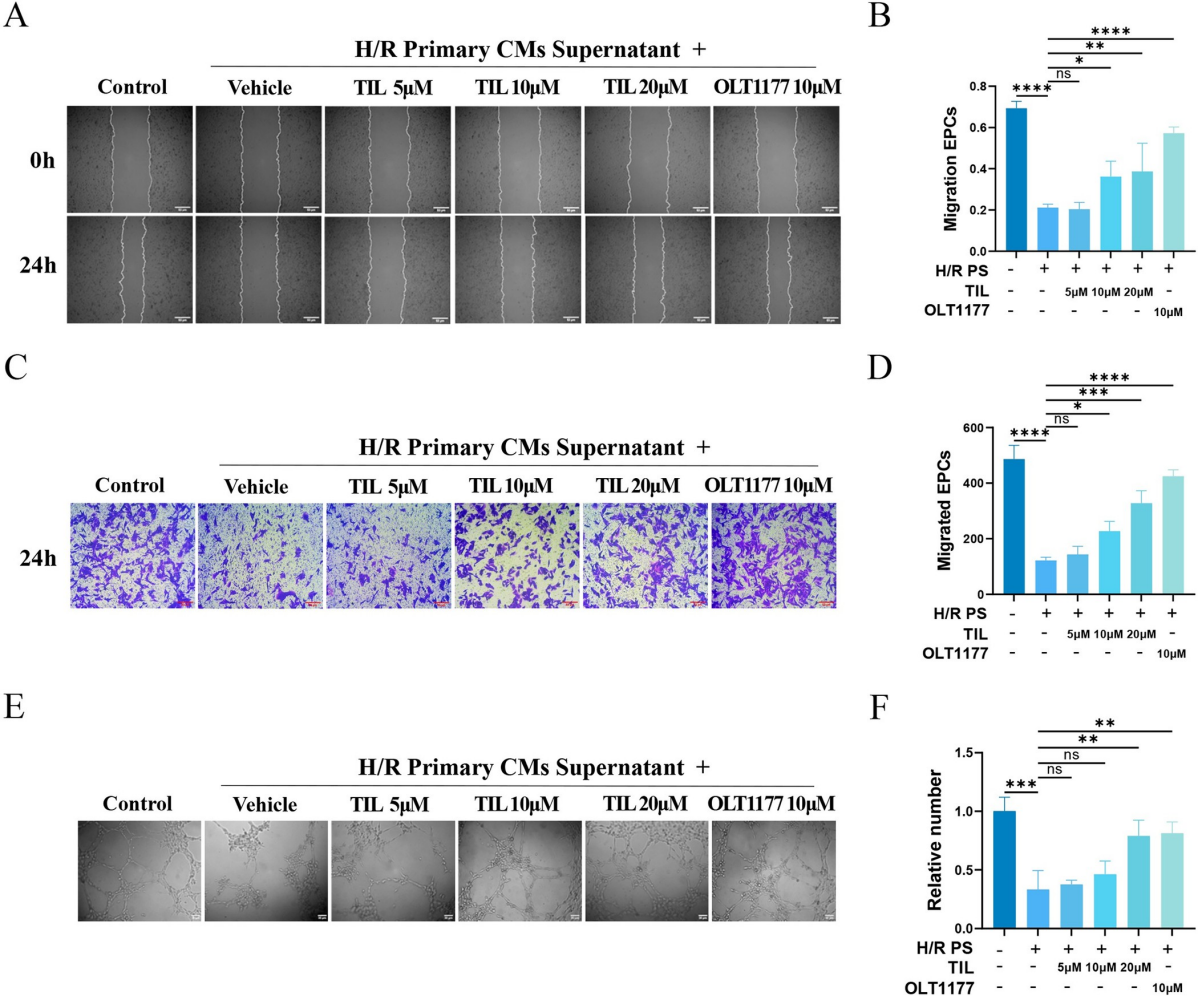
TIL improves migration and tube-forming ability of EPCs. EPCs were treated with different concentrations of TIL (5, 10, and 20 μM) or OLT1177 (10 μM) for 24 h before induction of EPCs with H/R PS for 24 h. H/R PS represents supernatant from H/R primary cardiomyocytes. (A, B) Representative micrographs of the invasiveness of EPCs in the scratch assay. The mobility of EPCs was calculated using Image J. (C, D) Representative micrographs of invasiveness of EPCs in transwell assay, and the migration of EPCs quantified by Image J. (E, F) Representative micrographs of invasiveness of EPCs in vascular-like structure formation assay. The tube formation of EPCs in each group was observed by tube formation method, and the number of tube formation in EPCs was quantitatively analyzed. Results are represented as mean ± SD. * denotes *P* < 0.05, ** *P* < 0.01, *** *P* < 0.001, **** *P* < 0.0001.

Building on the observed inhibitory effect of TIL on the Nlrp3 inflammasome, we next investigated TIL’s impact on the neovascularization capacity of EPCs. Our findings revealed that MIRI significantly reduced the migratory ability of EPCs. In contrast, TIL treatment counteracted this impairment in a concentration-dependent manner, enhancing the migratory ability of EPCs as evidenced by the results of the scratch wound assay (Fig 6A and 6B) and transwell migration assays (Fig 6C and 6D). tube-forming assays demonstrated that EPCs exposed to H/R primary cardiomyocyte supernatants tended to aggregate in sheets or remain flat on the Matrigel, failing to form normal tubular structures. However, EPCs treated with various concentrations of TIL were able to form complete tubular shapes (Fig 6E and 6F).

These results indicate that TIL can concentration-dependently enhance both the migration and tube-forming abilities of EPCs following stimulation with H/R primary cardiomyocyte supernatant, thereby promoting the functional capabilities of EPCs. Taken together with the aforementioned findings, we propose that TIL improves the neovascularization potential of EPCs by inhibiting the Nlrp3 inflammasome.

### 3.7 Cardioprotective effects of TIL-pretreated EPCs

**Fig 7:**
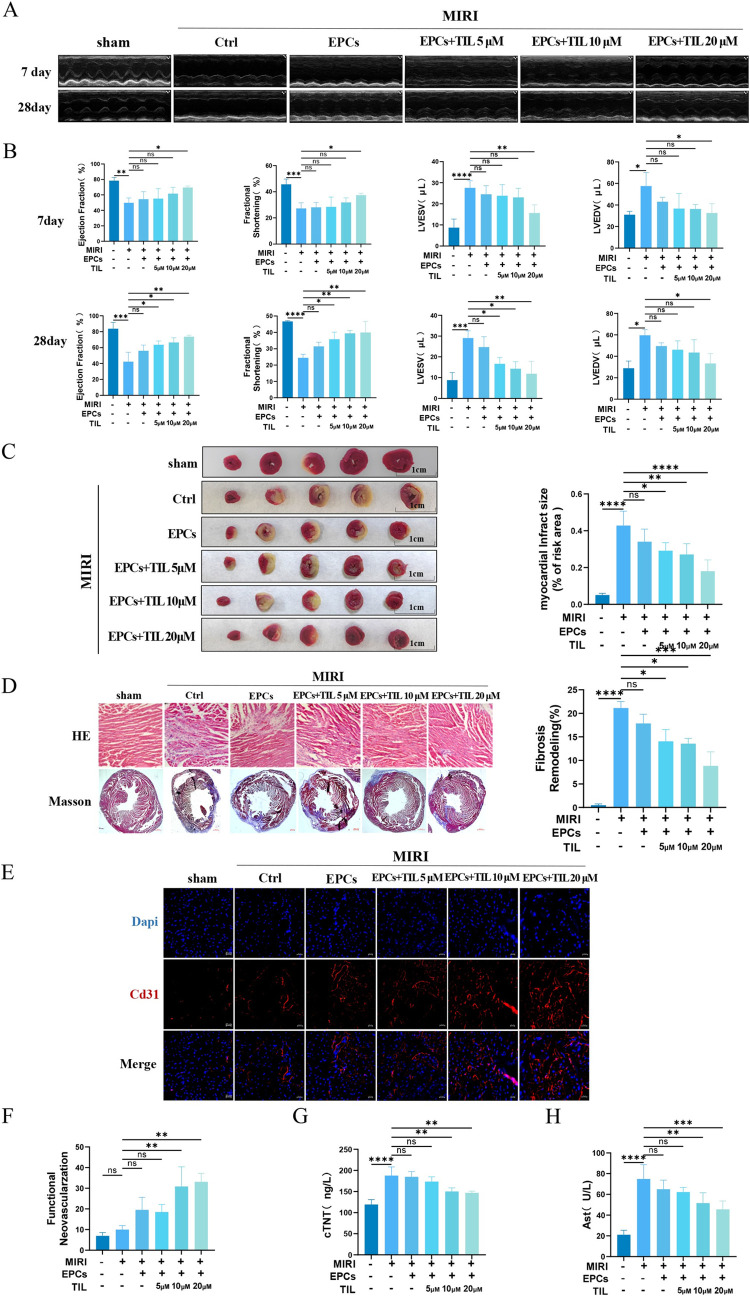
EPCs treated with different concentrations of TIL were given to improve cardiac function in MIRI mice. (A-C) Echocardiograms evaluating cardiac function including LVEF, LVFS, LVEDV and LVESV were performed on the 7 and 28 days after myocardial I/R. (D-E) Representative images of the heart after TTC staining, with non-infarcted tissue stained red and infarcted tissue stained white. The infarcted area (white) of the five TTC-stained sections was divided by the total area (red) to calculate the infarcted area (%) of I/R hearts. (F) Histopathological analysis of paraffin-embedded cardiac tissue sections. HE staining showed that EPCs treated with different concentrations of TIL attenuated the histopathological damage of MIRI. (G) Masson’s trichrome staining results showed that myocardial collagen fibers stained blue and cardiac myofibers stained red. EPCs treated with different concentrations of TIL reduced fibrosis. (H-I) Fluorescence images and quantification of functional neovascularization shown by microvessel density (Cd31). (J-K) Aspartate transaminase (Ast) and cardiac troponin T (cTnT) levels. Results are represented as mean ± SD. * denotes *P* < 0.05, ** *P* < 0.01, *** *P* < 0.001, **** *P* < 0.0001.

Building on our previous in vitro study of Nlrp3 inflammasome-mediated neovascularization in EPCs promoted by TIL, we proceeded to validate these findings in vivo. First, we successfully established a MIRI mouse model, and EPCs preconditioned with different concentrations of TIL were injected intramyocardially prior to reperfusion. We observed significant cardiac insufficiency in MIRI mice, as indicated by reduced LVEF and LVFS, alongside increased LVEDV and LVESV. These dysfunctions were markedly ameliorated in the MIRI+EPCs+TIL (5, 10, and 20 μM) groups (Fig 7A and 7B). TTC staining revealed a significant reduction in myocardial infarct size in the MIRI+EPCs+TIL (5, 10, and 20 μM) groups (Fig 7C). Histological analyses, including HE and Masson trichrome staining, were conducted to assess myocardial tissue morphology. HE staining results showed disorganized cardiomyocyte arrangement and significant inflammatory cell infiltration in the MIRI group, which were substantially improved in the MIRI+EPCs+TIL (5, 10, and 20 μM) groups (Fig 7D). Masson staining indicated elevated collagen fiber content in the MIRI group, while the MIRI+EPCs+TIL (5, 10, and 20 μM) groups demonstrated a significant reduction in collagen fiber content (Fig 7D). These findings suggest that TIL-pretreated EPCs significantly mitigate the pathological damage induced by MIRI. To further evaluate the protective effect of TIL on myocardial microvascular endothelial cells, we examined neovascularization during treatment with EPCs+TIL (5, 10, and 20 μM).

Immunofluorescence analysis revealed enhanced neovascularization in the TIL (20 μM) pretreated EPCs group, indicating significant proangiogenic potential (Fig 7E and 7F). Additionally, myocardial ischemia led to cardiomyocyte damage, as evidenced by serum enzyme levels. Serum Ast and cTNT levels were significantly higher in the MIRI group, reflecting severe cardiac impairment. However, these levels were notably reduced in the MIRI+EPCs and MIRI+EPCs+TIL groups, with the most significant effect observed in the latter (Fig 7G and 7H).

These findings suggest that TIL-pretreated EPCs have a protective effect against cardiac injury following MIRI, which can be attributed to the ability of TIL pretreatment to inhibit the inflammatory response associated with Nlrp3 inflammasome activation in EPCs. Previous studies have reported that TIL attenuates high-fat, high-carbohydrate (HFHC)-induced liver inflammation by inhibiting Nlrp3 inflammasome activation. Synergistic cardioprotective effects of TIL and syringin in diabetic cardiomyopathy through the interaction between the TLR4/NF-κB/NLRP3 and PGC1α/SIRT3 pathways have also been documented. Taken together, our study provides compelling evidence that TIL ameliorates MIRI injury and promotes neovascularization by inhibiting Nlrp3 inflammasome activation in EPCs.

## 4 Discussion

Ischemic heart disease (IHD) has long been a threat to human life and is considered the leading cause of death and disability worldwide. With the development and innovation of several therapeutic options, the prognosis of ischemic heart disease has improved significantly. However, MIRI remains a challenge in clinical practice, which can increase myocardial infarct size, myocardial fibrosis, and exacerbate cardiac dysfunction [46]. In the clinical studies reported thus far, the treatment of MIRI has primarily relied on supportive interventions, as no specific targeted therapies have been established [47]. Stem cell therapy has garnered significant attention in recent years, particularly the potential application of mesenchymal stromal cells (MSCs) in MIRI treatment, as documented in the literature [47]. Additionally, there have been numerous clinical reports on the use of CD34+ cell therapy for treating cardiovascular diseases [48,49]. A recent study by Jun Xie et al. highlighted that CD34+ is a widely expressed cell surface marker in various stem/progenitor cells, including EPCs and hematopoietic stem cells. CD34+ stem cells play a crucial role in cardiac remodeling following ischemia/reperfusion injuries, as they give rise to endothelial cells with high proliferative capacity, which may support post-infarction angiogenesis and local microcirculatory recovery, thereby limiting infarct size to some extent [50]. Furthermore, bone marrow-derived EPCs have been reported to contribute to neovascularization as well as the protection and repair of ischemic myocardium [51,52]. This aligns with the role of EPCs in promoting neovascularization after MIRI, as observed in our study. The NLRP3 inflammasome has gained considerable attention as a molecular switch that regulates inflammation. Numerous studies have indicated that myocardial injury is closely associated with NLRP3 inflammasome activation, which induces inflammatory responses [53], contributing to MIRI [54]. Moreover, NLRP3 has been reported to regulate EPCs function [34]. Yaping Deng et al. found that NLRP3 inflammasome activation contributes to EPCs dysfunction, while inhibiting caspase-1 activation and IL-1β formation aids in the functional recovery of EPCs. Furthermore, fenofibrate has been shown to improve the function of impaired EPCs via the NLRP3 pathway, thereby promoting angiogenesis [34]. onsistent with these findings, our study revealed that MIRI promotes Nlrp3 inflammasome activation, leading to EPCs dysfunction. In recent years, herbal medicine research has surged, and TIL has been extensively studied for its therapeutic effects and mechanisms across various diseases due to its diverse pharmacological activities. For instance, TIL has been reported to attenuate hepatic inflammation in non-alcoholic fatty liver disease (NAFLD) by inhibiting NLRP3 inflammasome activation [55]. Similarly, TIL has been shown to reduce inflammatory responses in diabetic retinopathy through the Nrf2/TXNIP/NLRP3 inflammasome pathway [56]. However, the relationship between TIL and the NLRP3 inflammasome in MIRI has not been previously explored.

In our study, we first confirmed the involvement of the Nlrp3 inflammasome in MIRI by detecting significant expression of Nlrp3 and Il-6 using qRT-PCR as inflammatory markers. Western blot analysis revealed that the H/R environment activated the Nlrp3 inflammasome and triggered the expression of inflammatory factors, which was notably reduced after Nlrp3 silencing. This aligns with previous reports on the role of the Nlrp3 inflammasome in regulating MIRI. Additionally, we found that the Nlrp3 inflammasome influences the mobility and angiogenic capacity of EPCs. In vivo validation demonstrated that silencing Nlrp3 in EPCs significantly improved cardiac function in MIRI mice, reduced infarct size, and mitigated pathological injury. Therefore, we propose that modulating the Nlrp3 inflammasome in EPCs may ameliorate MIRI. Subsequently, we simulated the MIRI environment in vivo using H/R. Western blot assays showed that TIL inhibited the expression of Nlrp3, Il-1β, and caspase 1-p20 in a concentration-dependent manner. It also inhibited the expression of Gsdmd, reduced ASC oligomerization, and decreased spot formation. These results are consistent with the inhibitory effects of TIL on the Nlrp3 inflammasome as reported in the literature. OLT1177 (dapansutrile), a potent and selective inhibitor of the Nlrp3 inflammasome in vivo and in vitro [57,58], produced effects similar to those observed with TIL in our study. The ameliorative effect of TIL on EPCs dysfunction due to Nlrp3 inflammasome activation was further validated in EPCs functional assays. Through in vivo efficacy validation, we found that TIL-pretreated EPCs improved cardiac function, reduced myocardial infarction area, and alleviated pathological injury in MIRI mice. Cd31 fluorescence expression was significantly enhanced after intracardiac injection of TIL-pretreated EPCs. Thus, our study demonstrates that TIL can improve EPCs dysfunction by modulating Nlrp3 inflammasome activity, thereby promoting neovascularization in MIRI.

EPCs derived from bone marrow, are pluripotent stem cells that have garnered significant interest for their potential in treating ischemic diseases. With the aim of restoring cardiomyocytes and cardiac function, stem cell-based therapies have been extensively examined in the past decade [59]. Although its promise, stem cell therapy faces challenges, including low survival rates of transplanted cells, inefficient homing to target tissues, risks of cardiac arrhythmias, potential for adverse immune reactions, and concerns about oncogenicity. TIL is a flavonoid compound, is widely utilized in managing cardiovascular and cerebrovascular conditions. Recent investigations into TIL have expanded, revealing its capability to safeguard against MIRI. This protective effect is attributed to its ability to counter oxidative damage and inflammatory responses, likely through its anti-endothelial dysfunction properties. Moreover, the scope of TIL research has broadened to encompass its potential benefits in managing conditions such as diabetes, hyperlipidemia, and atherosclerosis, highlighting its therapeutic versatility. In addition, TIL improves the function of EPCs through anti-inflammatory and other effects, thus expanding their applications.

## 5 Conclusions

The main findings of this study are as follows: (a) combined treatment with tilianin (TIL) and endothelial progenitor cells (EPCs) significantly ameliorated myocardial ischemia-reperfusion injury (MIRI) and promoted neovascularization; (b) TIL restored the functionality of damaged EPCs and inhibited the activity of the Nlrp3 inflammasome in the EPCs of MIRI-affected mice; (c) in vitro, TIL inhibited Nlrp3 inflammasome activity and reduced H/R-induced IL-1β expression, thereby reversing the dysfunction of H/R-injured EPCs and enhancing their neovascularization capacity. In summary, our study demonstrates that TIL can improve the function of damaged EPCs via the Nlrp3 inflammasome pathway, thereby accelerating the recovery of myocardial injury in MIRI mice. TIL also enhances EPCs function through its anti-inflammatory effects and other mechanisms, broadening its potential therapeutic applications. However, we were unable to assess the survival and homing efficiency of EPCs after intramyocardial injection. Additionally, the specific structural domains of TIL that target the Nlrp3 inflammasome remain unidentified and warrant further investigation.

## Supporting information

S1 Fig Immunofluorescence identification of primary cardiomyocytes. Dapi stained nuclei in blue, cTNT specific staining in green.
(TIF)

S2 Fig Immunofluorescence identification of primary endothelial progenitor cells. Nuclei stained for Dapi in blue and specifically for Cd133 and Cd34 in red.
(TIF)

S3 Fig Knockdown of *Nlrp3* in EPCs of WT mice. (A) Western blotting was performed to analyze the levels of Nlrp3 of EPCs. (B) Quantitative analysis of Nlrp3 gray values.
(TIF)

S4 Fig Graphical abstract. (TIF)

S1 Appendix Uncropped blots for immunoblot analyses. (PDF)

S1 File (DOC)

S1 Raw data (ZIP)
