# Supplementary figures and images for: Tilianin attenuates inflammasome activation in endothelial progenitor cells to mitigate myocardial ischemia-reperfusion injury
Source: PLoS One. 2024 Oct 10;19(10):e0311624. doi: 10.1371/journal.pone.0311624 (PMC11466386; doi:10.1371/journal.pone.0311624)

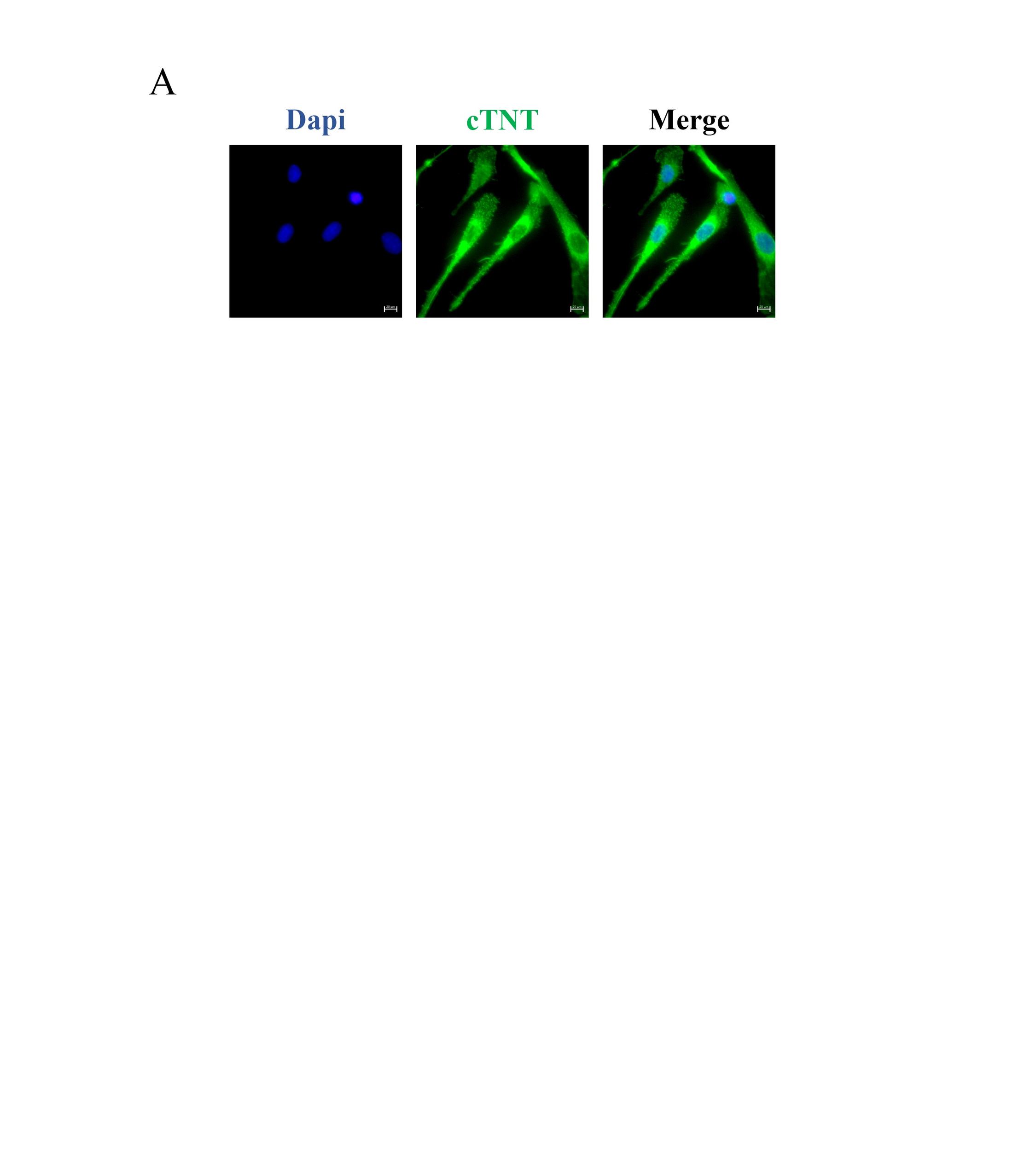

Supplement: S1 Fig — Dapi stained nuclei in blue, cTNT specific staining in green. (TIF) [file pone.0311624.s001.tif]

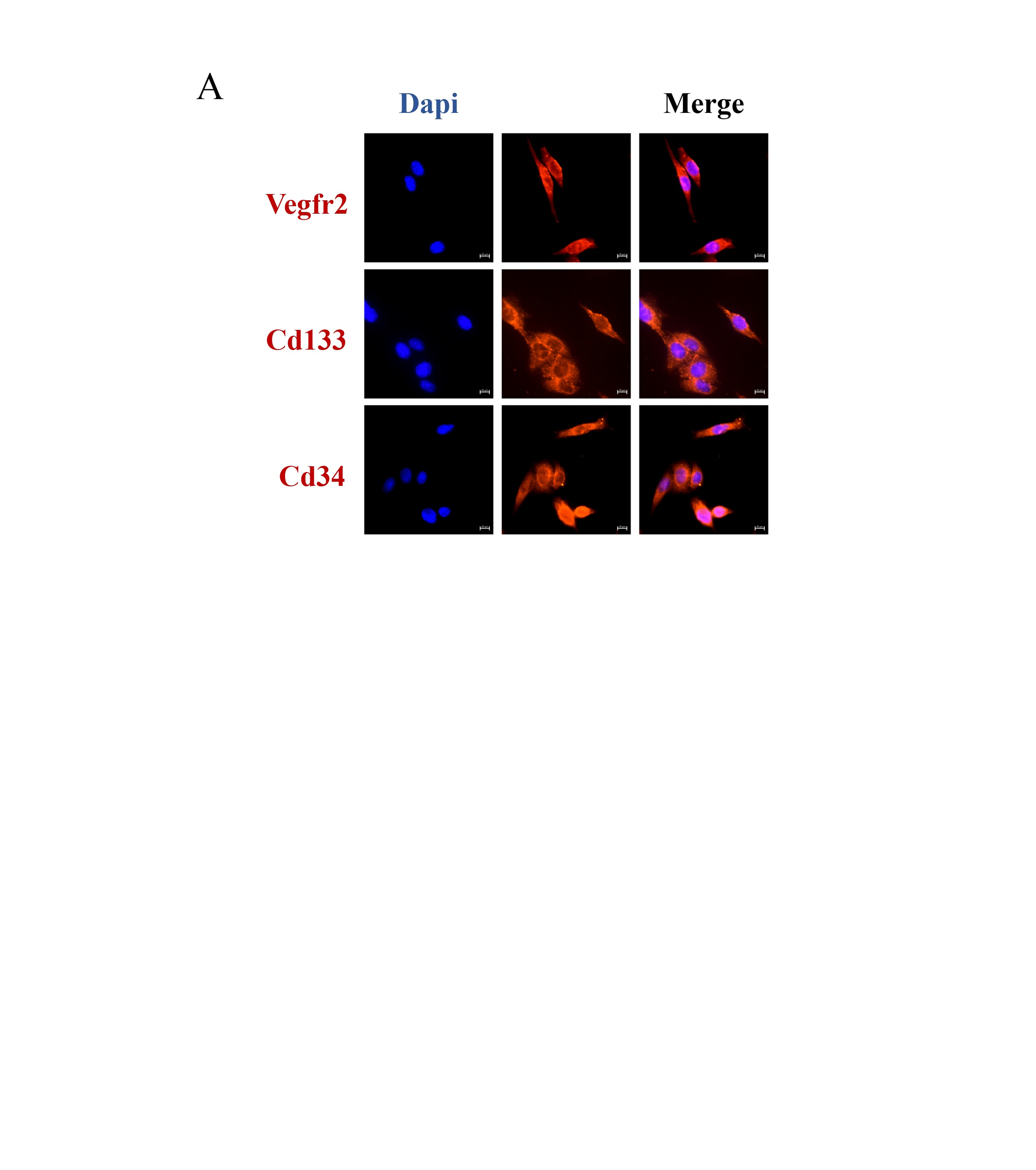

Supplement: S2 Fig — Nuclei stained for Dapi in blue and specifically for Cd133 and Cd34 in red. (TIF) [file pone.0311624.s002.tif]

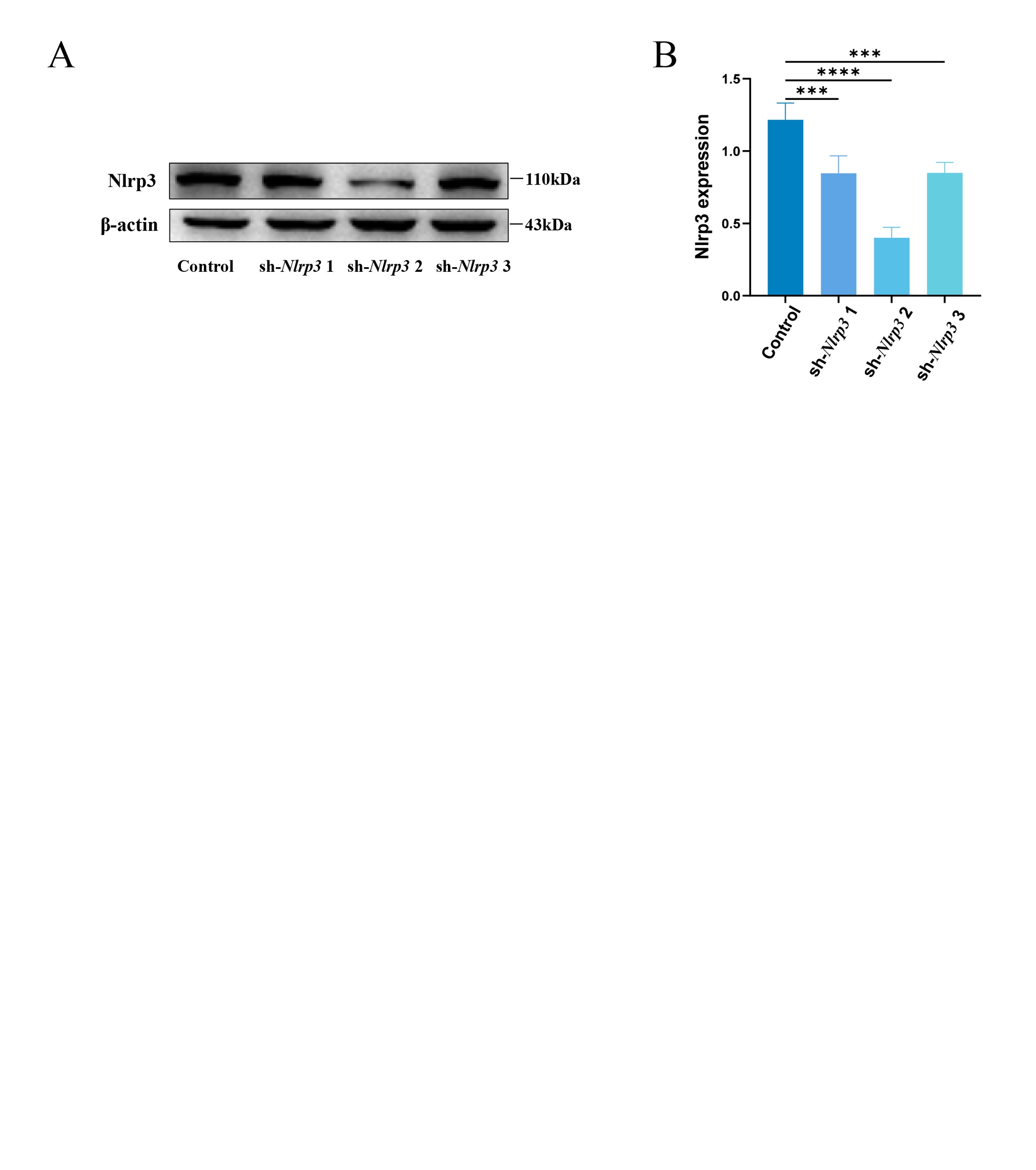

Supplement: S3 Fig — (A) Western blotting was performed to analyze the levels of Nlrp3 of EPCs. (B) Quantitative analysis of Nlrp3 gray values. (TIF) [file pone.0311624.s003.tif]

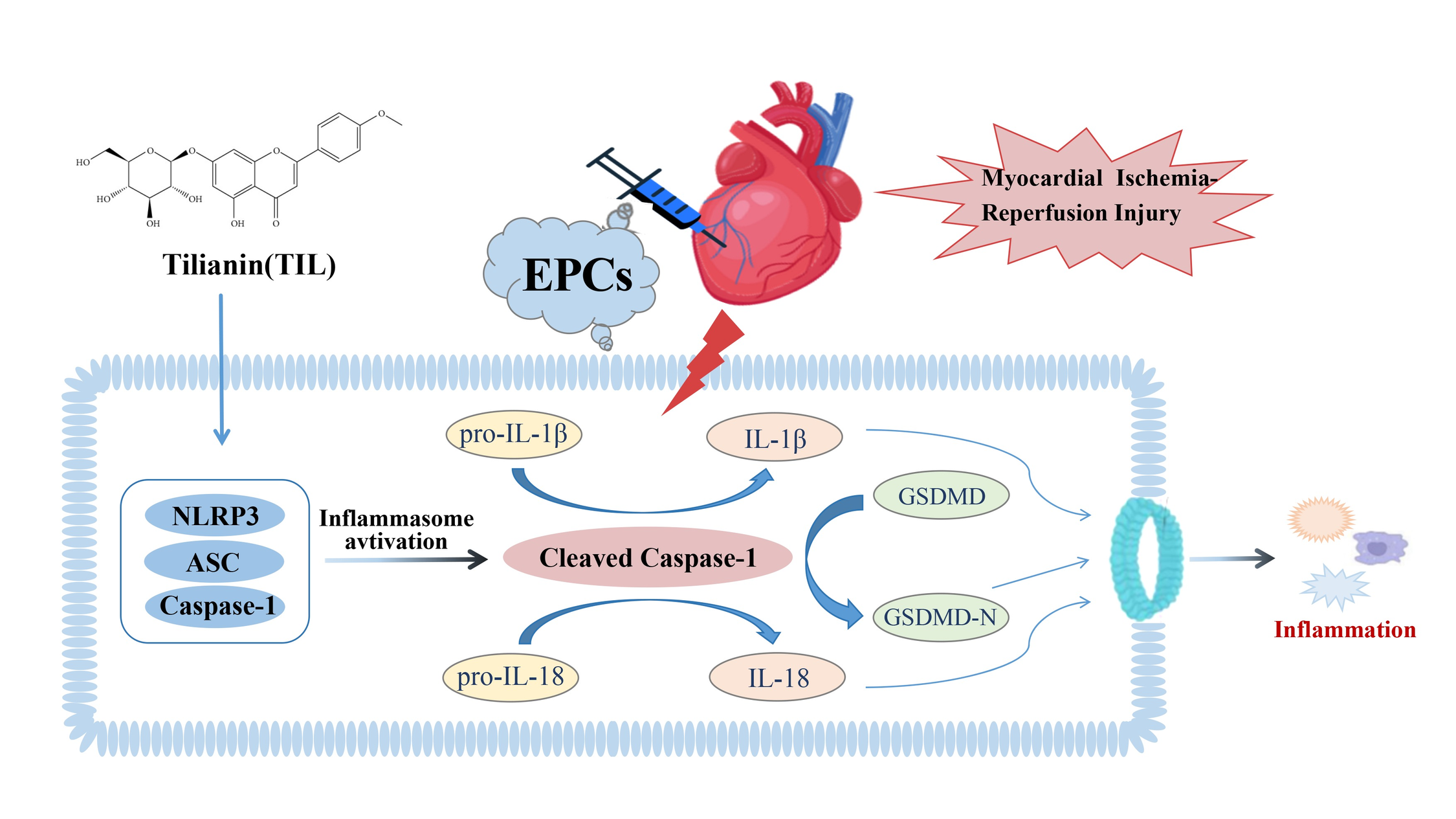

Supplement: S4 Fig — (TIF) [file pone.0311624.s004.tif]

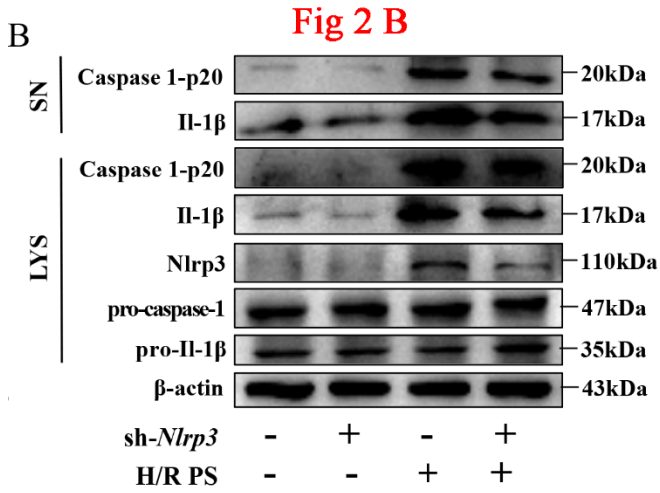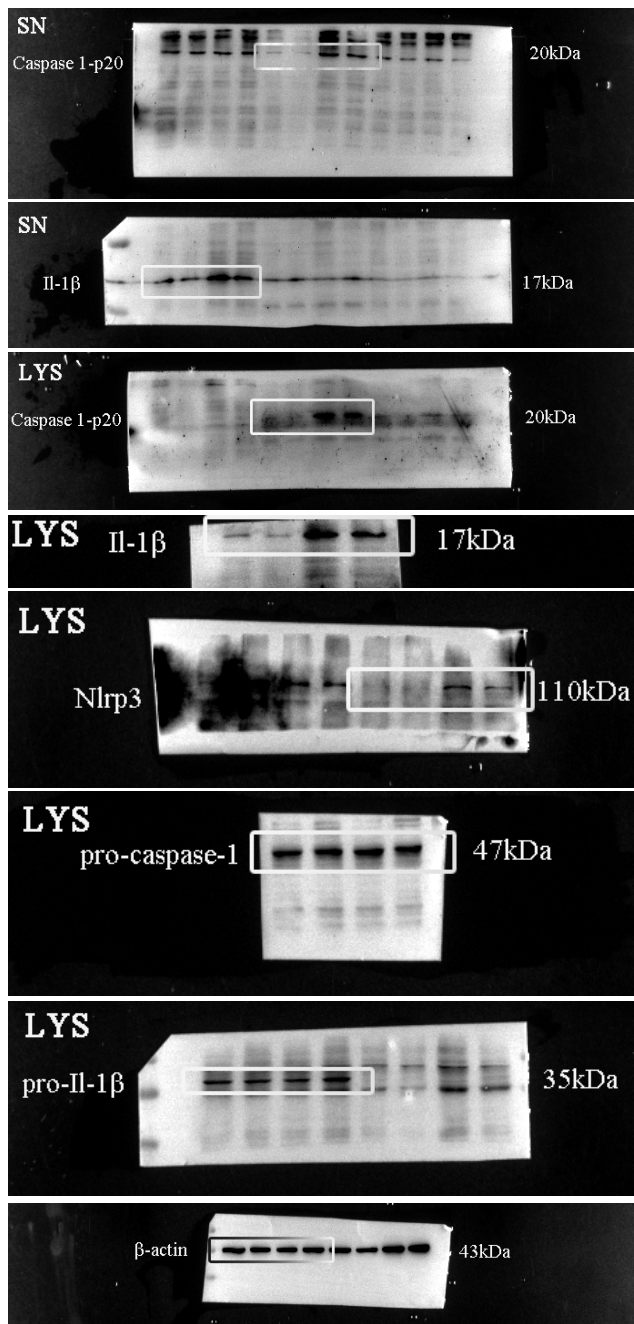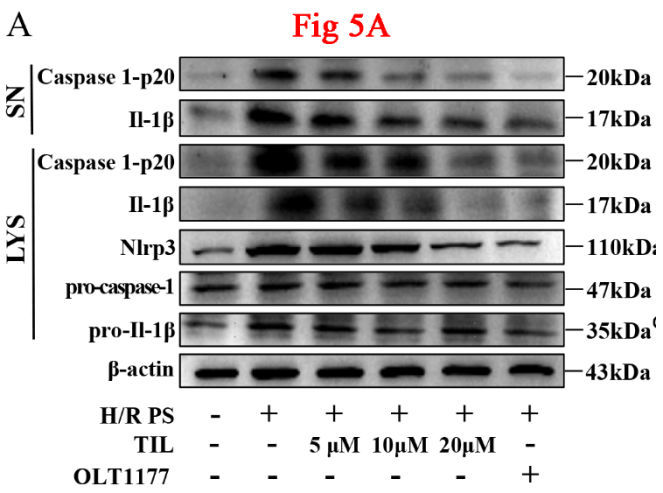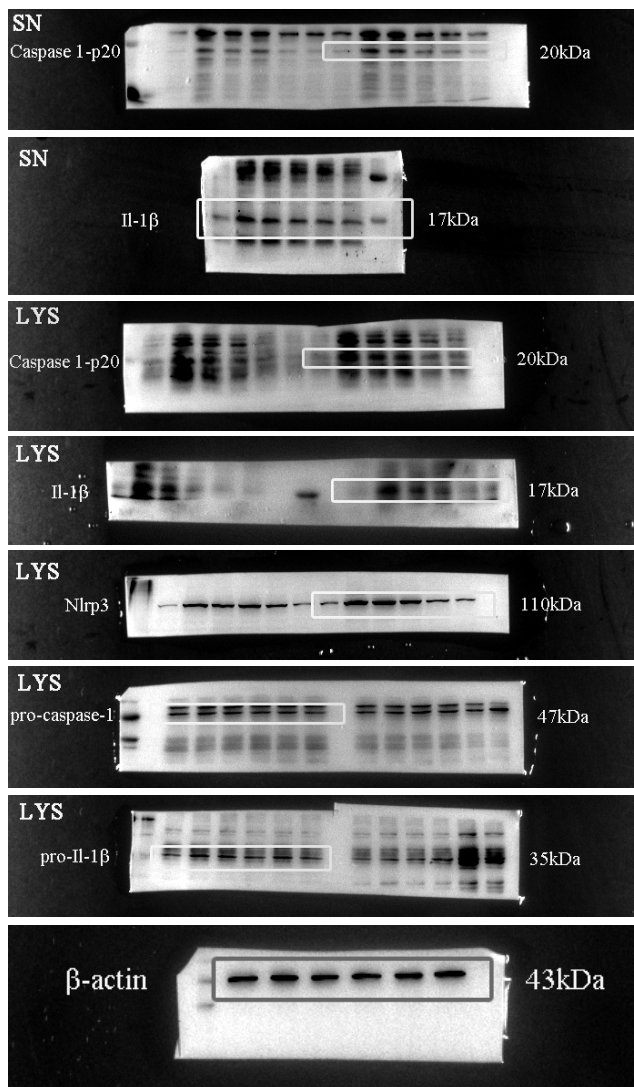

B

Fig 5 B

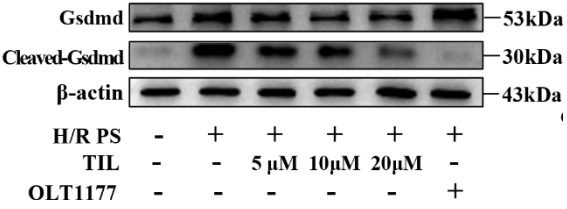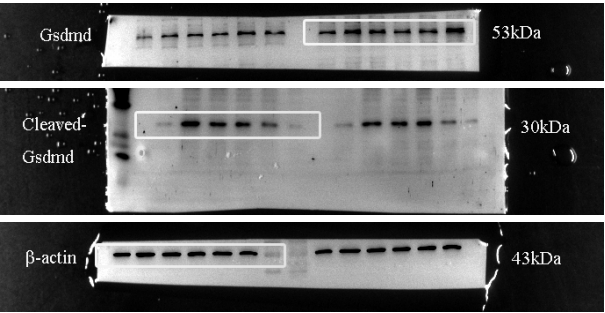

I

Fig 5 I

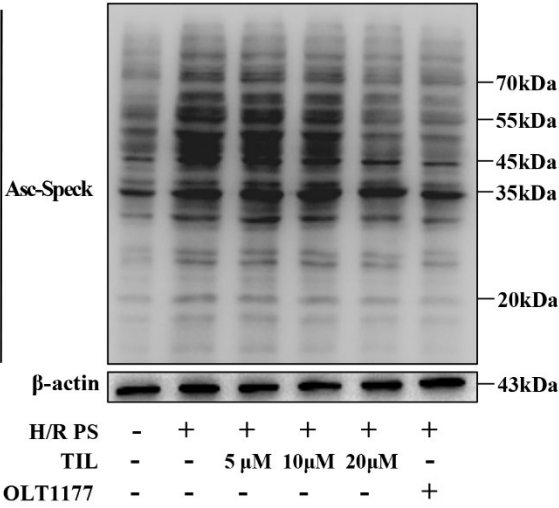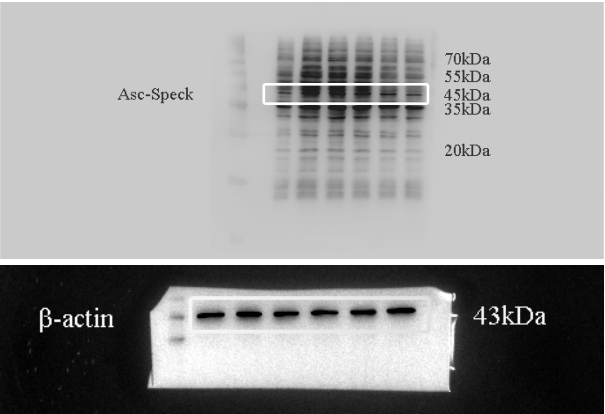

A

Fig S3

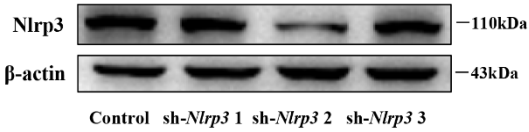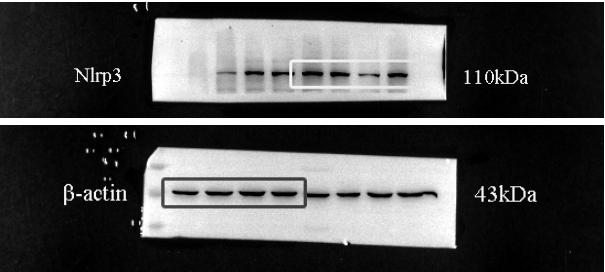

Supplement: S1 Appendix — (PDF) [file pone.0311624.s005.pdf]
