# Supplementary material for: Tilianin attenuates inflammasome activation in endothelial progenitor cells to mitigate myocardial ischemia-reperfusion injury
Source: PLoS One. 2024 Oct 10;19(10):e0311624. doi: 10.1371/journal.pone.0311624 (PMC11466386; doi:10.1371/journal.pone.0311624)
Supplement: S1 File — (DOC) [file pone.0311624.s006.doc]

**Supplementary Material**

# Cardiomyocyte cell isolation, culture and characterization

Adult mouse cardiomyocytes (ACMs) were isolated from 8-week-old adult mouse as previously described[1, 2]. Briefly, adult mouse hearts were extracted and mounted on a Langendorff device on a sterile operating table. The hearts were then perfused with calcium-free perfusion buffer (113 mM NaCl, 4.7 mM KCl, 0.6 mM KH_2_PO_4_, 0.6 mM Na_2_HPO_4_, 1.2 mM MgSO_4_, 10 mM Na-HEPES, 12 mM NaHCO_3_, 10 mM KHCO_3_, 0.032 mM phenol red, 30 mM taurine, 10 mM BDM, and 5.5 mM pH-7.0 glucose) for 5 minutes, followed by perfusion with 50 mL of digestion buffer (15,000 U type II collagenase and 50 μM CaCl_2_) for 10 min. Afterwards the hearts were cut into small pieces with ophthalmic scissors that would have been autoclaved and ground with a Pasteur pipette to isolate individual CMs. The cells were then centrifuged at low speed to remove the supernatant. The centrifuged CMs were spread in 6-well culture plates that had been well coated with fibronectin using F12 medium containing 10% FBS.

Regarding the identification of CMs in adult mice, in brief, CMs were inoculated in six-well plates at a suitable density. After cell attachment, the cells were fixed in paraformaldehyde for 15 min, then permeabilized with 0.3% (v/v) Triton X-100 (Solarbio, Beijing, China) in PBS for 15 min, blocked with 1% (w/v) bovine serum albumin (BSA, yuanye Bio-Technology Co., Shanghai, China) for 1 h, and then incubated with cTNT (1:200, Abways Technology, Inc., Shanghai, China) overnight, followed by incubation with 488-coupled goat anti-rabbit IgG (H + L) (1:200, Proteintech™, SA00009-2, Wuhan, China) for 2 h. Cell nuclei were stained with Dapi. Finally, fluorescence images were taken by a laser scanning confocal microscope (ZEISS Scope. A1, Germany). Image processing was performed using ZEN blue 2.3 software (Carl Zeiss, Germany). As (S1 Fig).

[Insert S1 Fig. near here]

**S1** **Fig.** **Immunofluorescence identification of primary cardiomyocytes.** Dapi stained nuclei in blue, cTNT specific staining in green.

# Hypoxia reoxygenation of primary cardiomyocytes

Mouse primary cardiomyocytes were taken and cultured in complete medium (DMEM high glucose medium, 10% FBS and 1% double antibody). After the cells were adhered to the wall, they were incubated in an anoxic chamber (95% N_2_, 5% CO_2_) for 22 h, and then continued to be incubated in a normal incubator with 5% CO_2_, 95% air, and 37 ℃ for 6 h. The supernatant of the cells was collected and centrifuged at 2000 rpm for 10 min, and then the supernatant was aspirated and stored.

# Isolation, culture and characterization of endothelial progenitor cells

Mice were decapitated and immersed in 75% alcohol for 15 min, and the femur and tibia were isolated in a sterile environment (care was taken not to cut the epiphyses as this would result in the loss of bone marrow fluid), washed with PBS, and the two epiphyses of the femur and tibia were clipped with heat-sterilized ophthalmic scissors. The bone marrow cavity was repeatedly rinsed with EBM-2 medium (2% FBS) until whitening. Next, the bone marrow fluid was filtered through a 70-mesh mesh filter and centrifuged at 250 ×g for 5 min, and the supernatant was discarded. Add 1 mL of erythrocyte lysate, then add 2 mL of PBS solution containing 2% FBS and lysed for 5 min at room temperature. Take a 15 mL centrifuge tube and add 3 mL of Histopaque-1083 (protected from light) and slowly add lysed bone marrow fluid along the wall of the tube and place it on top of the layer of fluid. The solution was centrifuged at 400 ×g for 20 min, and the solution was divided into three layers, the white translucent floc in the middle layer was rich in monocytes, the floc in the middle layer was aspirated and added to 10 mL of PBS containing 2% FBS to resuspend the cells, and then centrifuged at 250 ×g for 10 min, and the supernatant was discarded. The operation was repeated twice. Add EBM-2 complete medium to the cell sediment to resuspend the cells and add them to the 6-well culture plates which has been coated with fibronectin, and then put them into the carbon dioxide incubator for primary culture.

Regarding the identification of EPCs, briefly, EPCs from 7 days of primary culture were selected and inoculated in six-well plates at a suitable density. After 15 min of paraformaldehyde fixation, the plates were permeabilized with 0.3% (v/v) Triton X-100 (Solarbio, Beijing, China) in PBS for 15 min, blocked with 2% (w/v) bovine serum albumin (BSA, yuanye Bio-Technology Co., Shanghai, China) for 1 h, and then treated with Cd34, Cd133, and Vegfr2 (1:100, Proteintech™, Wuhan, China) overnight, followed by incubation with Cy3-coupled goat anti-rabbit (1:200, Proteintech™, SA00009-2, Wuhan, China) for 2 h. Cell nuclei were stained with Dapi. Finally, fluorescence images were taken by a laser scanning confocal microscope (ZEISS Scope. A1, Germany). Image processing was performed using ZEN blue 2.3 software (Carl Zeiss, Germany). As (S2 Fig).

[Insert S2 Fig. near here]

**S2 Fig.** **Immunofluorescence identification of primary endothelial progenitor cells.** Nuclei stained for Dapi in blue and specifically for Cd133 and Cd34 in red.

# Lentiviral vector construction, virus production, and infection

To knockdown *Nlrp3* expression in EPCs, sh-*Nlrp3* (pLKO.1-EGFP-Puro-Nlrp3-shRNA) or sh-NC (pLKO.1-EGFP-Puro-NC-shRNA) was co-transfected with the packaging plasmid psPAX2 and the envelope plasmid pMD2G, and viral supernatants were generated in 293T cells using Lipofectamine 8000 (Beyotime, Shanghai, China). Supernatants were harvested 48 h after transfection, filtered through Millex-HV 0.45 μm PVDF filters (Millipore, Billerica, MA), concentrated in PEG 8000, and stored at -80 ℃ for use.

To reduce the expression of *Nlrp3* in EPCs of WT mice, EPCs were infected with lentiviruses containing *Nlrp3* shRNA or nonsense shRNA, and we chose three target sequences for *Nlrp3* and nonsense sequences as follows:

*Nlrp3* shRNA 1: 5′-CCAGGAGAGAACCTCTTATTT-3′;

*Nlrp3* shRNA 2: 5′-CCGGCCTTACTTCAATCTGTT-3′;

*Nlrp3* shRNA 3: 5′-CCATACCTTCAGTCTTGTCTT-3′.

Nonsense shRNA: 5′-TTCTCCGAACGTGTCACGT-3′.

Transfection was performed according to the above procedure. After 48 h of transfection, western blot was performed to detect the expression of *Nlrp3*. Then the shRNA with the highest knockdown efficiency was selected. As (S3A-3B Figs).

[Insert S3 Fig. near here]

**S3 Fig.** **Knockdown of *Nlrp3* in EPCs of WT mice.** (A) Western blotting was performed to analyze the levels of Nlrp3 of EPCs. (B) Quantitative analysis of Nlrp3 gray values.

# Supplemental References:

1. Chen Y, Li X, Li B, Wang H, Li M, Huang S, et al. Long Non-coding RNA ECRAR Triggers Post-natal Myocardial Regeneration by Activating ERK1/2 Signaling. Molecular therapy : the journal of the American Society of Gene Therapy. 2019;27(1):29-45. Epub 2018/12/12. doi: 10.1016/j.ymthe.2018.10.021. PubMed PMID: 30528086; PubMed Central PMCID: PMCPMC6319349.

2. Huang S, Li X, Zheng H, Si X, Li B, Wei G, et al. Loss of Super-Enhancer-Regulated circRNA Nfix Induces Cardiac Regeneration After Myocardial Infarction in Adult Mice. Circulation. 2019;139(25):2857-76. Epub 2019/04/06. doi: 10.1161/circulationaha.118.038361. PubMed PMID: 30947518; PubMed Central PMCID: PMCPMC6629176.
